# Effectiveness and mechanisms of isometric resistance exercise to reduce blood pressure in a Chinese population: study protocol for a randomized-controlled trial

**DOI:** 10.1186/s13063-026-09798-x

**Published:** 2026-06-03

**Authors:** Sze Nok Ng, Benjamin Hon-Kei Yip, Regina Wing-Shan Sit, Kam-Sang Woo, Shuqi Wang, Daniel Dylan Cohen, Patricio Lopez-Jaramillo, Alexandre Persu, Guang Ming Tan, Véronique Cornelissen, Wook Bum Pyun, Babkowski Miguel Camafort, Samuel Yeung-Shan Wong, Eric Kam-Pui Lee

**Affiliations:** 1 The Jockey Club School of Public Health and Primary Care, The Chinese University of Hong Kong, Hong Kong, China; 2 Faculty of Medicine, Department of Medicine and Therapeutics, The Chinese University of Hong Kong, Hong Kong, China; 3 Department of Physical Education and Sports Sciences, University of Limerick, Limerick, Ireland; 4 Medical School, Masira Research Institute, Universidad de Santander (UDES), Bucaramanga, Colombia; 5 Cardiology, Cliniques Universitaires Saint-Luc, Université Catholique de Louvain, Brussels, Belgium; 6 Department of Medicine and Therapeutics, The Chinese University of Hong Kong, Hong Kong, China; 7 Department of Rehabilitation Sciences, KU Leuven, Louvain, Belgium; 8 Ewha Womans University Medical Center, Seoul, Republic of Korea; 9 Hypertension Unit Internal Medicine Department, Hospital Clínic, University of Barcelona, Barcelona, Spain; 10 Physiopathology of Obesity and Nutrition Networking Biomedical Research Centre, Carlos III Health Institute, Madrid, Spain

**Keywords:** Isometric resistance exercise training, Ambulatory blood pressure measurement, Chinese, Wall squat, RCT, Hypertension

## Abstract

**Background:**

Isometric resistance exercise training (IRT) involves sustained muscle contractions without changes in muscle length and joint angle, such as wall squats. Patients with hypertension may prefer IRT because they are easy to learn, require no equipment, are convenient, time-efficient, and can be performed anywhere. However, IRT have not shown to reduce blood pressure (BP) as detected by ambulatory BP measurements (ABPM), the reference standard for BP measurement. This study aims to examine the effectiveness of an IRT program in reducing BP.

**Methods:**

This randomized controlled trial (RCT) will involve 390 patients with a suboptimal daytime systolic BP of > 135–160 mmHg on a 24-h ABPM, who do not meet the current World Health Organization recommendations for physical activity. Participants will be randomly assigned to either the IRT (wall squat) group or stretching exercise (active control) group (1:1), using stratified and blocked randomization according to age (< 45, 45– < 60, and ≥ 60 years old), sex, and anti-hypertensive drug use (users versus non-users). Investigators will be blinded to randomization sequence and allocation. IRT group will receive a well-structured, widely accepted, and validated 24-week wall squat program (2 min per exercise, 2 min of rest between sets, and 3 sessions per week). Adherence will be monitored by regular patient communication via social media. The control group will receive the same treatment, with the exercise replaced by frequency-matched and time-matched stretching exercise. The primary outcome measure will be systolic daytime ABPM at 24 weeks. Secondary outcome measures will include other BP and ABPM parameters at 12 weeks, 24 weeks, and 12 months, carotid-femoral pulse wave velocity at 24 weeks and 12 months, and flow-mediated dilation at 24 weeks and 12 months. Safety data will be collected and reported.

**Discussion:**

This trial will be the first adequately powered RCT to determine whether IRT can be recommended as a treatment for hypertension, involving a Chinese population. It will also contribute new knowledge regarding the mechanisms of IRT. This research has the potential to influence clinical practices. If positive results are obtained, this research could potentially alleviate the substantial burden on healthcare systems caused by poor BP control.

**Trial registration:**

ClinicalTrials.gov NCT06510998. Registered on July19, 2024.

**Supplementary Information:**

The online version contains supplementary material available at 10.1186/s13063-026-09798-x.

## Protocol version {2}

Study protocol version 1 dated July 23, 2024.

## Introduction

### Background and rationale {9a}

Hypertension (HT) is the most prevalent chronic condition and is the leading cause of cardiovascular and renal morbidity and mortality [1]. Despite effective treatments, only approximately one-third of patients with HT have optimal control of their blood pressure (BP) [1]. Although anti-HT medications can effectively reduce BP, patients’ non-compliance to medications is prevalent globally (~ 40%), due to misconceptions (e.g., medications can cause renal damage) and psychosocial factors (e.g., forgetfulness) [2]. Similarly, patients have poor adherence to the effective aerobic and dynamic resistance exercises because additional time, skills, training venue, and equipment are required [3]. Conversely, isometric resistance exercise training (IRT), involving low intensity, sustained isometric exercise, can overcome these barriers because they are safe, time efficient (approximately 14 min per session, 3 sessions per week), easy to learn, and require little to no equipment [4]. Isometric exercise involves a muscle contraction with no change in muscle length and joint angle, contrasting with dynamic resistance exercise which involves repeated muscle shortening and lengthening. In clinical practice and research, the two most well-studied and well-structured IRT are the wall squat and hand grip exercise [4, 5]. Furthermore, when compared with other types of exercise, IRT carry no joint impact (lower risk of joint injury), have lower myocardial oxygen demand (less chest pain or ventricular arrhythmias and more suitable for patients with cardiovascular diseases), and require less space (more suitable for patients living or working in crowded areas or with limited mobility) [6, 7]. The latest 2023 network meta-analysis also found IRT most effective to reduce office BP (by ~ 8/4 mmHg) among all exercise modalities (e.g., aerobic/resistance training) in reducing office BP (by ~ 8/4 mmHg) [8].

An individual study demonstrated that IRT can effectively reduce 24-h BP on ambulatory BP monitoring (ABPM) [9]. However, a 2023 meta-analysis showed that IRT have not reduced 24-h BP or daytime BP on ABPM in patients with HT despite reducing office BP [5]. Although IRT have reduced night-time BP on ABPM, the results were highly heterogeneous and imprecise (systolic BP [SBP]: − 4.28 mmHg, I2 = 72.7%) [5]. As that meta-analysis described, the lack of definite improvement on ABPM has prevented wide implementation of IRT for HT patients because ABPM is consistently more reproducible, more predictive to cardiovascular outcomes and is the “gold” standard of BP measurements [5, 10, 11]. It is therefore unclear whether IRT truly reduced patients’ BP or only minimized white-coat effects. Currently, international guidelines recommended aerobic and resistance training and commented a lack of evidence for IRT [12, 13]. Furthermore, the anti-HT effects of IRT have been understudied in the Chinese population, and the recent systematic review has not identified any Chinese study [5, 14]. However, cardiovascular responses to exercise vary amongst ethnicities [15]. Because Chinese patients are more salt sensitive and physical exercise can reduce salt sensitivity, IRT may be more effective in reducing BP in Chinese [16, 17]. Wall squats may also be more implementable in Chinese because many of their daily activities are conducted in a squatting positing (i.e., the Asian squat) [18].

Furthermore, the mechanisms of BP reduction by IRT remain unclear. For instance, IRT (including wall squats) are hypothesized to partially occlude arteries (e.g., in the thigh and legs) by sustained muscle contractions and stimulate flow-mediated dilation (FMD) of vessels once the muscle contractions end. Therefore, progressive and repetitive exposures to IRT may enhance nitric-oxide mediated vasodilation and vessel diameters [5]. Although vascular changes and reduction in peripheral vascular resistance were hypothesized to be the main mechanisms of IRT, FMD or vascular stiffness (as measured by pulse wave velocity [PWV]) has yet shown to change after IRT in a 2023 meta-analysis due to limited studies [14]. Although IRT have been shown to improve heart rate variability (as an surrogate of autonomic nervous system activities), no included studies in the above two 2023 meta-analyses studied BP dipping status and 24-h BP variability (BPV), which are independent predictors to cardiovascular events and death [11, 19]. As BPV and BP dipping are determined by autonomic nervous system activities and vascular stiffness, these predictors to cardiovascular events may be improved by IRT [20, 21].

There are also important limitations in the existing relevant randomized-controlled trials (RCTs). (i) Although wall squat exercise may have superior hypotensive effect than hand grip, only 1 existing wall squat RCT (*n* = 48, lasted 4 weeks) measured ABPM [5]. (ii) The 4 existing relevant ABPM studies included in the 2023 meta-analysis were small, time-limited (≤ 12 weeks) and underpowered (*n* = 20 to 48, total *n* = 147), and recruited exclusively participants with normal ABPM BP (mean daytime BP < 135/85 mmHg) [5]. An ongoing Brazilian RCT (NCT03896334) lasting for 24 weeks is evaluating handgrip exercise rather than wall squat as in the present study. Third, as advocated by latest reviews, most RCTs had flaws in blinding of the assessors and allocation concealment, and no RCTs had monitored activities in the control group [5, 14]. Four, safety data is under-reported [4].

### Explanation for the choice of comparator {9b}

In the study design, stretching exercise was chosen for comparator. Previous meta-analysis showed that passive static stretching exercises did not change BP level in most RCTs and therefore can be active control in this RCT [22]. While there was no widely validated sham comparator or standardized control for isometric wall squat in RCTs, the use of passive static stretching exercises as a comparator will ensure that participants in both intervention and control groups perform some form of frequency-matched and time-matched interventions. This active control reduces potential bias related to passive controls. Details of the designs of control group is described in the Intervention and comparator section.

### Objectives {10}

The aim of the study is to investigate the effectiveness of the IRT in reducing BP of Chinese adults with sub-optimal BP, defined as a suboptimal daytime SBP of > 135–160 mmHg.

The objectives are to compare the effectiveness of wall squat exercise (intervention) and stretching (active control) for the following measures:

1. To determine whether a 24-week IRT program leads to a reduction in daytime ABPM SBP compared to an active control group (primary objective)
2. To assess the effects of IRT on 24-h, nighttime, and office BP at 12 weeks, 24 weeks, and 12 months after recruitment. This will provide data on the persistence of long-term daytime ABPM SBP reduction achieved through IRT at 12 months after recruitment
3. To assess the effects of IRT on ABPM BP variabilities at 12 weeks, 24 weeks, and 12 months after recruitment
4. To investigate the persistence of daytime ABPM SBP reduction achieved through IRT at 12 months after recruitment
5. To evaluate whether IRT achieves BP reduction by improving vascular stiffness (measured through PWV) at 24 weeks and 12 months and endothelial function (measured through FMD) at 24 weeks and 12 months. Besides being potential mechanisms underlying BP reductions, PWV and FMD are also independent predictors to cardiovascular events [23, 24].
6. To assess the safety and tolerability of IRT among patients with HT

## Methods: patient and public involvement, and trial design

### Patient and public involvement {11}

We conducted a pilot RCT on IRT, which included interviews exploring participants’ experience concerning the IRT intervention and trial design. The findings are used to modify the research question, study design, and recruitment and implementation phases.

### Trial design {12}

**Fig. 1:**
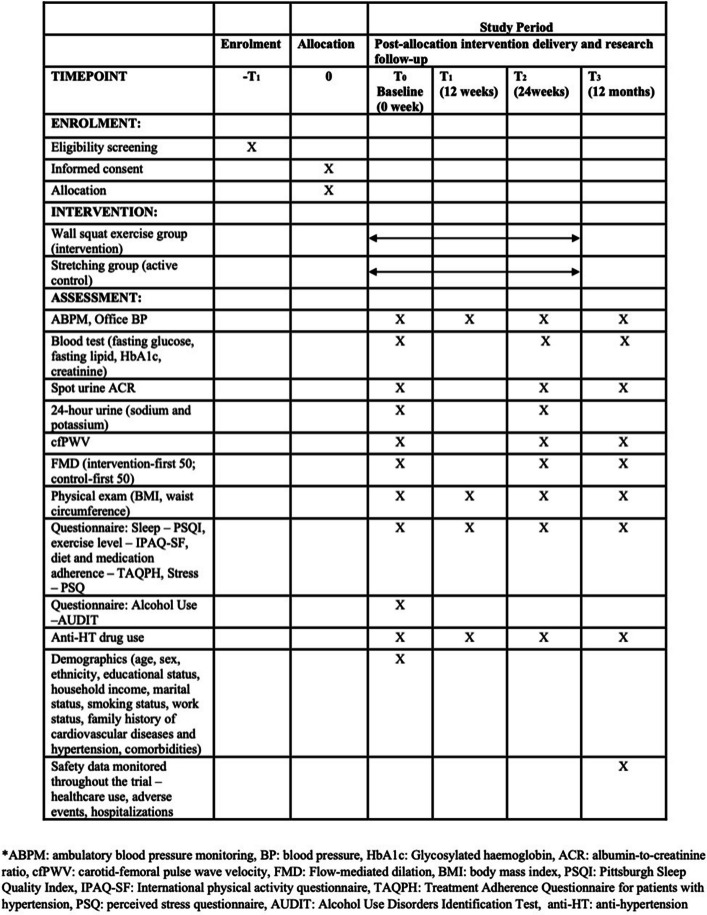
Schedule of enrollment, interventions, and assessments

This protocol was written with reference to the Standard Protocol Items: Recommendations for Interventional Trials (SPIRIT) [25]. The SPIRIT checklist can be found in additional document 1. A parallel-arm (IRT vs. stretching exercise [active control]) RCT will be conducted in a 1:1 ratio. The primary objective is to determine whether an IRT program leads to a reduction in daytime ABPM SBP compared to an active control group at 24 weeks. Further data collection will be conducted at 12 weeks and 12 months post-recruitment to ensure safety and investigate long-term effects of the IRT program respectively (Fig. 1). Meditating and moderating roles of patients’ characteristics and secondary outcomes (e.g., FMD/PWV) to BP reduction by IRT will be investigated. The RCT was pre-registered on clinicaltrials.gov (NCT06510998) and was approved by the Joint Chinese University of Hong Kong-New Territories East Cluster Clinical Research Ethics Committee (2022.618). The study complies with the Declaration of Helsinki.

### Trial setting {13}

Participants will be recruited from the general public and primary care in Hong Kong by (i) direct recruitment of patients with HT by doctors/nurses and posters when they attended the publicly funded family medicine clinics (FMCs); and (ii) newspaper advertisements.

### Eligibility criteria for participants {14a}

Patients meeting all these criteria will be included: (i) a suboptimal daytime SBP of > 135–160 mmHg on a 24-h ABPM (the internationally recommended cut-off for elevated BP, the rationale for focusing on SBP is explained under “Outcomes”) [12]; (ii) reported no regular physical activity or less than that recommended for adults by the World Health Organization (e.g. < 150 min of moderate-intensity aerobic exercise per week); (iii) on stable doses of anti-HT medication(s) for ≥ 4 weeks if the patient is receiving drug treatments; (iv) agree for no drug changes during the intervention period (24 weeks); and (iv) aged ≥ 18 years old. Patients receiving or not receiving anti-HT drugs will be included to maximize external validity and investigate responders’ characteristics. Only patients with mildly elevated daytime SBP of > 135–160 will be included to ensure safety as drug changes are avoided as far as possible during the 24-week intervention period. Patients will be excluded if any of the following criteria are met: (i) cannot provide informed consent; (ii) unwillingness to repeat ABPM (primary outcome); (iii) relative contraindications to ABPM (i.e., diagnosed atrial fibrillation, night-time workers, occupational drivers, or patients with bleeding tendencies); (iv) severe osteoarthritis pending knee replacement surgery (these patients may be unable to perform the IRT (i.e., wall squat exercise)); (v) known secondary HT; (vi) use of ≥ 3 anti-HT medications at maximum doses or ≥ 4 anti-HT medications (these patients have resistant HT and represent another disease spectrum); (vii) SBP or Diastolic BP (DBP) are > 160 mmHg or > 100 mmHg, respectively, on ABPM at baseline or at 12 weeks follow-up to ensure safety (these patients require early treatment); (viii) pregnancy/breastfeeding; and (ix) active malignancy (Table 1).

**Table 1:** Inclusion and exclusion criteria

**Inclusion criteria**	
- A suboptimal daytime SBP of > 135–160 mmHg on a 24-h ambulatory blood pressure monitoring	
- No regular physical activity or less than that recommended for adults by the World Health Organization (< 150 min of moderate-intensity aerobic exercise per week)	
- On stable doses of anti-HT medication(s) for ≥ 4 weeks if the patient is receiving drug treatments	
- Agree for no drug changes during the intervention period (24 weeks)	
- Aged ≥ 18 years old	
**Exclusion criteria**	
-Cannot provide informed consent	
-Unwillingness to repeat ambulatory blood pressure monitoring (primary outcome)	
-Relative contraindications to ambulatory blood pressure monitoring (i.e. diagnosed atrial fibrillation, nighttime workers, occupational drivers, or patients with bleeding tendencies)	
-Severe osteoarthritis pending knee replacement surgery (these patients may be unable to perform the IRE (i.e. wall squat exercise))	
-Known secondary hypertension	
-Use of ≥ 3 anti-hypertensive medications at maximum doses or ≥ 4 anti-hypertensive medications (these patients have resistant hypertension and represent another disease spectrum)	
-Systolic blood pressure or diastolic blood pressure are > 160 mmHg or > 100 mmHg, respectively, on ambulatory blood pressure monitoring at *baseline or at 12 weeks follow-up to ensure safety* (these patients require early treatment)	
-Pregnancy/breastfeeding	
-Active malignancy	

### Eligibility criteria for sites and those delivering interventions {14b}

Exercise techniques will be taught prior to beginning the program after randomization. Both initial exercise training and outcome data collection will take place at the Lek Yuen Family medicine clinic (FMC) located in Shatin, Hong Kong only. After that, participants will be remotely monitored as they perform home-based wall squat exercises or passive static stretching, according to their randomized group.

### Who will take informed consent? {32a}

Following the eligibility screening, written informed consent will be obtained by trained research assistants.

### Additional consent provisions for collection and use of participant data and biological specimens {32b}

Participants will be asked to provide optional consent for the storage of the spot urine sample at CU-Med Biobank for future studies.

## Intervention and comparator

### Intervention and comparator description {15a}

#### Intervention arm: remotely monitored home-based self-learnt wall squat exercise

Each exercise session will contain 4 sets of 2-min wall squat isometric holds with 2 min of rest between each set (approximately 14 min per session in total), and a total of 3 sessions will be arranged every week (with ideally 48 h between exercises); this is the most commonly used and most researched structure that has been shown to reduce BP [4, 5]. Our 24-week program is the longest among existing RCTs (mean = 9.8 weeks) [4]. While the longest study of wall squat, lasting a year, it included only 24 patients and did not use ABPM [26]. Although previous studies used an incremental isometric wall squat test to determine exercise intensity (i.e., knee angle during the exercise) for each patient, this is labor intensive and not feasible for routine clinical practice and large RCTs [9, 27]. It will, therefore, reduce the external validity of our study for clinical implementation. Conversely, our co-investigators, DDC and PL-J, used an alternative standardized algorithm to stratify patients to one of two starting joint angles, followed by standardized progression of intensity (reduction in joint angle) every 2–3 weeks. They found a similar SBP reduction at 12 weeks using this algorithm (i.e., 12.9 mmHg), an improvement comparable with other studies using initial incremental tests in non-medicated patients with HT [28]. Furthermore, the standardized algorithm had excellent patient acceptability, with no dropouts and 100% attendance in the wall squat group [28] (Table 2). A slightly modified version of this algorithm will be used in the current RCT by incorporating the validated rating of perceived exertion scale, which allowing individualized exercise progression towards 95° by 10 weeks (the maximum possible intensity) (Tables 2 and 3) [29]. This allows more capable patients to progress early if the exercise intensity is inadequate (i.e., recurrent rating of perceived exertion of < 4) [29]. This approach aims to balance the individualization of exercise intensity progression and the need to deliver an adequate stimulus while considering the patient’s comfort, motivation, and safety. Wall squat exercise techniques and the use of rating of perceived exertion will be taught prior to beginning the program. This includes the algorithm in Table 2 (i.e., progressive knee angles) and posture (feet are shoulder width apart, back and head in a neutral position/against the wall). This will be reinforced by written materials and a video (which times the exercise and reminds the patient of exercise techniques). Patients will be asked to videotape themselves performing the wall squat at the 2nd or 3rd week. They will send this to the investigators via a messaging app (e.g., WhatsApp) who will review to identify and where appropriate, correct error in execution of the exercise.

**Table 2:** Prescription of wall squat to participants

Baseline	Can wall squat at 95’ for 2 min*	Cannot squat at 95’ for 2 min
Week 1	125’	135’
Week 2	125’ (115’)	135’ (125’)
Week 3	125’ (115’)	125’ (115’)
Week 4	115’ (105’)	125’ (115’)
Week 5–6	115’ (105’)	115’ (105’)
Week 7–9	105’ (95’)	105’ (95’)
> Week 10	95’	95’

^*^If the patient has rating of perceived exertion of < 4 at end of 2 consecutive sessions, they can increase the intensity of the exercise early as shown in the ()

**Table 3:** Validated rating of perceived exertion

Rating	Description	Time to failure
0	Rest	100%
1	Extremely easy	90%
2	Very easy	80%
3	Easy	70%
4	Somewhat easy	60%
5	Moderate	50%
6	Somewhat hard	40%
7	Hard	30%
8	Very hard	20%
9	Extremely hard	10%
10	Maximal	0%

Adherence to exercise and physical activity level will be monitored using a smart watch, which will record exercise type and duration and the heart rate during the isometric exercises. Data from the smart watch will be examined by the research assistant weekly and patients will be contacted if the session of that are missed. Furthermore, as there will be change of knee angle every 1–2 weeks, the assistant will check the knee angle and safety at least every 2 weeks via patients’ preferred messaging app (e.g., WhatsApp) and answer any questions they might have. The patient will be asked standardized questions (e.g., “Using the perceived exertion scale, what’s the difficulty level on your last weeks’ wall squat?”, “Is there any problem performing the exercise, including discomfort or injury?”, “What is the knee angle that you will be using in the coming week?”). Participants will be asked to refrain from beginning new exercises (i.e., those not part of their prior usual habits) during the 24-week study period. Although the intervention has been well accepted by patients with HT in our previous research [28], we also successfully conducted another pilot study in 12 Chinese patients with HT from August to November 2022 using an abbreviated version of this exercise program (8 weeks instead of 24 weeks) to evaluate feasibility. We found that repeated ABPMs are acceptable to patients and there was > 87% can fully adhere to the exercise program. No adverse event was reported by patients.

#### Control arm: stretching exercise

Although stretching exercise was used in another IRT RCTs (NCT03896334), there was no widely validated sham control for wall squat in RCTs. Previous meta-analysis found that passive static stretching exercises did not change BP level in most RCTs and therefore can be active control in this RCT [22]. A frequency-matched (3 ×/week) and time-matched (~ 14 min each session) passive static stretching exercise will be used (a sample in the additional document 2 based on previous RCT using stretching and had no hypotensive effects) and taught to patients prior to beginning the program (with video support as in IRT group). The treatment in the control group, such as the research assistant’s contact, the use of smart watch and monitoring of adherence, will be the same as in the intervention group. This approach was suggested by our reviewers last year to control for hypotensive effects due to frequent contact by the research team and feedback from the smart watch in the IRT group.

#### Criteria for discontinuing or modifying allocated intervention/comparator {15b}

Participants are encouraged to adhere to their assigned allocation as closely as possible. However, if they experience serious illnesses (e.g., a traffic accident resulting in limb loss or severe infections that immobilize them) that prevent them from performing the prescribed exercises (such as wall squats or stretching), the exercises will be discontinued. We will encourage these participants to provide outcome data as far as possible. Participants also have the option to withdraw from the study at any time.

#### Strategies to improve adherence to intervention/comparator {15c}

To encourage participants to maintain their intervention habits, the research assistant will review the exercise diary weekly to bi-weekly and contact participants if their exercise frequency is insufficient during the 24-week intervention period. After this initial phase, the diary will be checked monthly, and the research assistant will contact participants if their exercise remains inadequate, until the final data collection at 12 months.

#### Concomitant care permitted or prohibited during the trial {15d}

While medical consultations and intake of medication are allowed, change in hypertensive medications should be avoided as far as possible during the 24-week intervention period.

#### Harms {17}

There are no anticipated risks associated with the wall squat and stretching exercises, as these interventions have been found to be safe, and adverse events are rare [4]. However, if any suspected or actual complications arise, participants are instructed to seek medical assistance, including attending the accident and emergency unit (access is not limited by current trial), and to contact the study team at their earliest convenience. As part of adherence monitoring, all participants already have the contact information for the research team. Any hospitalizations will be promptly reported to the relevant ethics committee within 24 h, or as soon as possible.

#### Ancillary and post-trial care {34}

Although no additional ancillary or post-trial care will be provided by the research team, the trial is designed to help participants in both the intervention and control groups establish new exercise habits. These habits may lead to sustainable, home-based exercise routines that can be maintained after the intervention. Otherwise, all participants will continue their follow-up with their usual health service providers. Clinical trial insurance has been purchased to cover compensation for any participants who experience harm as a consequence of participation in this study.

#### Participant timeline {18}

The schedule of enrolment, interventions, and assessments is summarized in Fig. 1.

#### Sample size {19}

Table 4 demonstrates the required sample sizes to identify a true difference in SBP reduction between the two intervention groups, with various assumptions on the true difference and type I error (beta). Type II error is fixed at 0.05. Based on previous reviews, SBP reduction by IRT ranged from approximately 3 to 8 mmHg [5]. A change of 3 mmHg is considered minimal clinically meaningful effect [30]. We therefore use the most conservative SBP difference of 3 mmHg between the 2 arms, and use a standard deviation of approximately 10 mmHg (on ABPM), which is typical in the HT population and confirmed in our previous local studies [10]. Targeting a statistical power of 0.8 and assuming a dropout rate of 10%, we are aiming to recruit a total of 390 participants, with 195 participants in each group [9]. This sample size is adequate if the true BP difference is 4 mmHg and at a power of 0.9 (Table 4).

**Table 4:** Required sample size

Power (1-beta)	True mean reduction in SBP reduction between intervention groups (Intervention-Control group)*			
	3 mmHg	4 mmHg	5 mmHg	6 mmHg
0.80	175	99	63	44
0.85	200	113	72	50
0.90	234	132	85	59

^*^The required sample size is based on the targeted type I error (beta) and assumed true mean reduction in SBP between treatment groups while type II error is fixed at 0.05

#### Recruitment {20}

The research team has developed strategies to ensure adequate enrollment and achieve the target sample size. For instance, the recruitment rate will be regularly monitored monthly. Using newspaper advertisements will already ensure that our recruitment reach participants across the entire territory of Hong Kong.

## Assignment of interventions: randomization

### Sequence generation: who will generate the sequence {21a}

The randomization sequence will be generated by an independent statistician, using the statistic program “Random Allocation Software,” version 1.1.0.

### Sequence generation: type of randomization {21b}

Despite yet conclusive, a systematic review suggests that age and anti-HT drug treatments may affect response to IRT [31]. As sex-specific difference in HT is increasingly emphasized and investigated, sex is considered an important factor in stratification randomization [32]. Therefore, stratified randomization with a randomized block size of 4 or 6 will be utilized according to age (< 45, 45– < 60, and ≥ 60 years old [a cut-off used in the above review]), sex, and anti-HT drug use (users versus non-users).

### Allocation concealment mechanism {22} and implementation {23}

The randomization sequence will be printed out, and sealed in sequential and light-opaque envelopes, which will only be opened after a participant’s consent form is signed. The envelops will be kept in a closed locker to which only research assistants will have access. The envelopes will only be opened by participants after written informed consent is obtained by research assistants. The envelopes will then be filed in participant’s folder.

## Assignment of interventions: blinding

### Who will be blinded {24a}, how will be blinding be achieved {24b}, and procedure for unblinding if needed {24c}

Blinding of participants is not possible after randomization owing to the nature of the intervention. However, the investigator, outcome assessor(s), and statistician will be blinded.

## Outcomes, data collection, and management

### Outcomes {16}, plans for assessment, and collection of outcomes {25a}

After data collection at baseline, participants will return for outcome assessments at 12 weeks, 24 weeks, and 12 months. Details of the time point for outcomes and trial instruments are specified in each outcome below. All research assistants who are involved in data collection will be trained the correct use of instruments (e.g., ABPM) and questionnaires, as well as retrieval of data. After each assessment, the files will be reviewed to identify missing or erroneous data. Any missing information will be retrieved immediately from the instruments and the study participants.

### Primary outcome: daytime SBP assessed via ABPM at 24 weeks (immediate post-intervention)

Mean SBP will be the primary outcome since it is more predictive of cardiovascular outcomes than DBP, especially in older adults [33]. WatchBP O3 (by Microlife) is a validated and recommended ABPM monitor by multiple international societies (available at https://www.stridebp.org/bp-monitors) and will thus be used in this trial. ABPM will be conducted on recruitment and at 12 weeks, 24 weeks (primary study endpoint), and 12 months. IRT session will be separated from ABPM measurement by at least 24 h [34]. ABPM will measure the BP every 30 min [35]. The actual sleep time will be estimated by a sleep diary, which is the preferred method as shown in our previous research and by international recommendations [36]. The methods for conducting ABPM (including the use of the non-dominant arm and appropriate cuff size) and the definition of a valid ABPM report will be in accordance with the latest European Society of Hypertension ABPM measurement guidelines [37]. Our prior research experience showed that patient adherence to repeated ABPMs can be poor because sleep may be affected by ABPM in some patients. Therefore, although wearing the ABPM monitor for at least 24 h will be encouraged, ABPM will be performed only during waking hours in reluctant patients whose sleep is affected [37]. This will not affect our primary outcome.

### Secondary outcomes

Other ABPM parameters, which also predict cardiovascular outcomes, will be our secondary outcomes and include mean daytime SBPs at other time-points (i.e., 12 weeks and 12 months), mean daytime DBP, mean nocturnal SBP/DBP, mean 24-h SBP/DBP, BP dipping status, and BP variability (by average real variability as recommended by systematic review) at all time-points (12 weeks, 24 weeks, and 12 months) [38].

### Carotid–femoral pulse wave velocity

Carotid–femoral pulse wave velocity (cfPWV) and pulse waves will be analyzed by applanation tonometry using SphygmoCor (AtCor Medical Pty Ltd.) with the accompanying SphygmoCor Software Suite (SphygmoCor System). SphygmoCor is the most well-validated device for measuring PWV [39]. The measurements will be obtained with the patient in a supine position after at least 5 min of resting, and the cfPWV will be recorded sequentially at the carotid and femoral artery. Although aortic PWV (aPWV) was suggested by the research council for another project, cfPWV measurement is recommended by international guidelines and the device for measuring aPWV (Mobil-O-Graph [IEM, Germany]) is not available in Hong Kong [23]. PWV is the gold standard for measuring large artery stiffness, is reversible after short-term interventions (e.g., 6–12 weeks), is an independent predictor of cardiovascular events, and has also been reduced after aerobic endurance exercise and low-to-moderate intensity resistance exercise training [40, 41]. cfPWV will be measured on recruitment and at 24 weeks and 12 months.

### FMD of brachial artery

The test is widely used clinically to assess endothelial function (i.e., endothelium-dependent vasodilation) in human as described previously [42–45]. In brief, FMD will be performed using a high-resolution linear array transducer (L10-5), with a median frequency of 7.5 MHz and a standard Sonosite System (model MicroMaxx, Bothell, USA). The brachial artery diameter will be measured manually, with due attentions of reproducibility, coefficient of variation, and a stringent FMD protocol, according to international guidelines (e.g., in a quiet, temperature-controlled room, subject lying supine, refraining from ingesting caffeine) [46, 47]. The longitudinal image of brachial artery is obtained 5–10 cm above the elbow. After a qualified image is acquired for analysis, a pressure cuff placed distal to the imaging probe at the forearm will be inflated to 50 mmHg above the SBP for 5 min. The longitudinal image of the brachial artery is scanned continuously for 3 min after the deflation of the cuff (reactive hyperemia). The percentage change in peak brachial artery diameter before and after the cuff inflation will be calculated and reported as the FMD of the subject. Due to budget constraints, FMD measurement cannot be provided to all participants. Instead, FMD measurement will be performed by a blinded senior sonographer with full training and practical sonographic experience for over 30 years [43–45], in a subsample of patients in the intervention group (*n* = 50) and in the control group (*n* = 50). FMD measurement will be conducted at baseline, 24 weeks, and 12 months.

### Patient safety

The safety of IRT is currently underreported in RCTs [4]. Therefore, adverse events (including emergency department visits and hospitalizations) and the number of FMC and private clinic visits, as well as the incidence of falls, musculoskeletal injuries, new-onset stroke, ischemic heart diseases (including angina and myocardial infarction), heart failure, renal failure, and deaths, will be self-reported and retrieved from the HK Hospital Authority computerized medical system (CMS). These will be monitored throughout the trial and at the last data collection endpoint (i.e., 12 months).

### Other cardiovascular risk factors

These are important parameters for predicting future cardiovascular events and deaths and will be monitored at baseline, 24 weeks, and 12 months. These include fasting serum glucose, glycosylated hemoglobin, fasting serum lipid profile (total cholesterol, high-density and low-density lipoproteins, and total triglycerides), serum creatinine, and spot urine albumin-to-creatinine ratio.

### Covariates that affect BP

Patients may change their behavior, knowing about their suboptimal BP. Physical activities will be monitored by smart watch above. Other lifestyle changes were assessed by validated methods. For instance, (i) sleep quality and duration as measured by the validated Pittsburgh Sleep Quality Index questionnaire; (ii) physical activity level and sitting time duration, by the validated International Physical Activities Questionnaire (short form); (iii) diet and medication adherence (if on anti-HT medications), by the medication (9 questions) and diet (9 questions) domains of the Treatment Adherence Questionnaire for Patients with Hypertension; and (iv) stress level, by the Perceived Stress Questionnaire [48–51]. Overweight and obesity will be evaluated by body mass index (body weight/height2) and waist circumference measurements. Anti-HT drug use data will be retrieved from the CMS (or self-reported if patient follow-up is not conducted in public clinics). These will be measured at baseline, 12 weeks, 24 weeks, and 12 months. Furthermore, sodium and potassium intake will be assessed by 24-h urine collection at baseline and 24 weeks (primary end-point).

### Baseline data collection

Data on age, sex, ethnicity, educational status, household income, marital status, smoking status (current smoker, ex-smoker, or never smoked), work status, family history of cardiovascular diseases and HT, and comorbidities (such as diabetes mellitus, hyperlipidemia, cardiovascular diseases [e.g., stroke, ischemic heart disease, heart failure], and chronic kidney disease) will be collected using the CMS and by self-reporting. Alcohol use will be assessed by the validated Alcohol Use Disorders Identification Test [52].

### Plans to promote participant retention and complete follow-up {25b}

Participants are given supermarket coupons after they completed data collection at each timepoints (i.e., baseline, 12 weeks, 24 weeks, and 12 months) to reinforce participant adherence to the trial and data collection. Also, regular communication will be maintained with participants to promote adherence to the trial and complete follow-up. Data of participants at all timepoints will be collected even after the withdrawal of the interventions, with their consents. For participants who discontinue the trial, reasons for withdrawal will be obtained.

### Data management {26}

The data will be entered into the computerized database weekly or bi-weekly, depending on the recruitment rate. Data entries will undergo a double-checking process by two independent assessors to ensure accuracy and reliability. Each participant will be assigned a unique identification code for research data management. The code list can only be accessed by the research team and will be securely maintained by the principal investigator after the study is completed. All data are securely stored in password-locked computers and locked cabinet. Personal information will remain confidential throughout the study and will not be disclosed in future publications.

### Confidentiality {33}

The confidentiality of sensitive data (i.e., personal information) will be ensured by minimizing the number of personnel who handles subject data. Data will also be kept in an encrypted folder, accessible only to the research team. Participant records will be stored in a locked cabinet. To further ensure confidentiality, each participant in the study will be assigned a unique identifying number for monitoring throughout the trial.

## Statistical methods

### Statistical methods for primary and secondary outcomes {27a}

The baseline characteristics of participants in the two arms will be described using proportions and means, as appropriate to the standard deviations. Our primary analysis will employ ANCOVA, with SBP at 24 weeks as the outcome variable, and SBP at baseline and intervention group as covariates. Secondary analyses will be exploratory and will include ANCOVA of secondary outcome measures, as well as linear mixed models (LMMs) to delineate changes in SBP trends within the same group at different time points. Utilizing a full-information likelihood approach, LMMs will incorporate all available data and effectively handle missing data, similar to multiple imputation methods.

The proportion of patients with adverse effects will be compared by chi-square tests. For primary analysis, we will not adjust unbalanced confounders, which are assumed balanced after randomization. In sensitivity analysis, potential unbalanced confounders at baseline will be added. Mediation will be used to explore how IRT is mediated through PWV/FMD to differentiate the indirect effect and direct effect. Moderation effect will be estimated by estimating the interaction between patients’ characteristics and IRT. Mediation and moderation analyses are exploratory since our sample size is targeted for our primary objective.

### Who will be included in each analysis {27b}

All randomized participants will be included in the analysis.

### How missing data will be handled in the analysis {27c} and methods for additional analyses (e.g. subgroup analyses) {27d}

In our funding proposal, we initially stated that “the analysis will be conducted on an intention-to-treat basis (primary analysis) and a per-protocol basis (sensitivity analysis).” However, per the European Medicines Agency (ICH E9[R1]), these terms can be ambiguous, and relevant estimands should be employed [53]. In this RCT, we anticipate intercurrent events (ICEs) such as changes in medications during the first 24 weeks, non-adherence to interventions, severe illnesses that prohibit participants to perform exercises (as described above), and switches between intervention groups. For our primary analysis, we will utilize the “treatment policy” estimand, which treats the occurrence of ICEs as irrelevant and uses the actual observed values for the outcome variable [53]. This approach aligns with our previous intention-to-treat analysis. For our secondary analysis, we will adopt the “hypothetical strategy” estimand, which estimates outcomes assuming all patients in their respective groups adhere perfectly to the interventions [53]. Additionally, missing data will be addressed through multiple imputation [54, 55].

### Interim analyses {28b}

An interim report will be submitted to General Research Fund, Hong Kong SAR, the funder of the current study (available at: https://www.ugc.edu.hk/eng/rgc/index.html). Clinical trial insurance was purchased to cover the compensation for participants who are harmed as a consequence of participation in this study.

### Protocol and statistical analysis plan {5}

In addition to publishing the protocol on an international journal for public access, the results of this RCT will be disseminated through an international peer-reviewed journal and international and local conferences. This is a trial funded by General Research Fund, a formal written request for accessing the de-identified data and code is required for consideration after the main manuscript is published.

## Oversight and monitoring

### Composition of the coordinating center and trial steering committee {3d}

A single research team will be responsible for overseeing the trial and there will be no coordinating center and trial steering committee. The research team will hold monthly study group meetings to discuss the progress of the trial and report any severe adverse events to the ethics committee.

### Composition of the data monitoring committee, its role, and reporting structure {28a}

Due to the low-risk nature of the study and that the funder did not allocate resources for a Data Monitoring Committee (DMC), this study will not have a DMC.

### Frequency and plans for auditing trial conduct {29}

Research team will hold monthly meeting on discussing the recruitment progress, demographic balances between two arms, data collection, and adverse events. Financial auditing of the current study will be performed by the Chinese University of Hong Kong SAR.

### Protocol amendments {31}

A formal amendment application will be submitted to ethics committee before implementation. If any changes needed to be made to the protocol, the relevant parts of the study, as well as the record in the trial registry, will be updated (ClinicalTrials.gov, NCT06510998).

### Dissemination policy {8}

The results of this RCT will be disseminated through an international peer-reviewed journal and international and local conferences.

## Discussion

### Potential importance for research

This is the first adequately powered RCT using wall squat. To provide long-term data, which is lacking among all except one RCT [26], the current RCT will collect primary outcome data at 24 weeks. This will balance scientific rigor with patients’ safety because anti-HT drug titration will be avoided as far as possible during the 24-week study period. The sample size is larger than all existing RCTs using ABPM combined (total *n* = 147). Although this project is adequately powered for the primary outcome (i.e., whether IRT can reduce daytime ABPM SBP), the size (USD 205,000) and duration (≤ 3 years) of the current funding preclude a long and large RCT that can detect a reduction in “hard” clinical outcomes such as cardiovascular events and mortality. Therefore, our expert team of researchers plan to conduct this RCT in the UK, Colombia, Spain, South Korea, and Belgium with respective local research funding (see the list of co-investigators). Furthermore, if the RCTs find a reduction of BP by IRT, we plan to apply for further funding to follow up these patients to detect cardiovascular events and mortality. The resultant data will be combined in a prospective individual patient data meta-analysis. Therefore, the current RCT is an essential component of an international, multi-center, and landmark trial with the potential to change clinical practice. The resultant large sample size will also allow investigation of responders’ characteristics (including ethnicity) and may help unravel the significant heterogeneity of results from existing RCTs [4]. This RCT will be the cornerstone for studies that will investigate cost-effectiveness and BP reduction mechanisms of IRT.

### Trial status

This trial is actively recruiting participants (190/390). This trial is ongoing and has a planned duration of 29 months. The recruitment began on 1 January 2025. The first participant was recruited in April 2025 and the estimated date of completion is 31 May 2027. The current protocol is Version 1 on July 23, 2024.

## Supplementary Information

Additional file 1. SPIRIT checklist 1.

Additional file 2. Passive and static stretching exercises (control group) 2.

## Data Availability

The data will be available after the main manuscript is published upon reasonable request.

## Abbreviations

HT: Hypertension
BP: Blood pressure
IRT: Isometric resistance exercise training
ABPM: Ambulatory blood pressure monitoring
FMD: Flow-mediated dilation
PWV: Pulse wave velocity
BPV: Blood pressure variability
RCTs: Randomized-control trials
SPIRIT: Standard Protocol Items–Recommendations for Interventional Trials
FMCs: Family medicine clinics
cfPWV: Carotid-femoral pulse wave velocity
aPWV: Aortic PWV
CMS: Computerized medical system
LMMs: Linear mixed model
SBP: Systolic blood pressure
DBP: Diastolic blood pressure
DMC: Data Monitoring Committee
ICEs: Intercurrent events
