# Supplementary material for: Effectiveness and mechanisms of isometric resistance exercise to reduce blood pressure in a Chinese population: study protocol for a randomized-controlled trial
Source: Trials. 2026 Jun 3;27:519. doi: 10.1186/s13063-026-09798-x (PMC13449400; doi:10.1186/s13063-026-09798-x)
Supplement: Supplementary file 2 [file 13063_2026_9798_MOESM2_ESM.docx]

**Additional document 2. Passive and static stretching exercises (control group)** (adopted from: Hotta K, Kamiya K, Shimizu R et al. Stretching exercises enhance vascular endothelial function and improve peripheral circulation in patients with acute myocardial infarction. Int Heart J 2013; 54: 59–63)

| The exercises consist of five components, with each component lasting 35 seconds and a 20-second rest in between. The total duration of the entire exercise is approximately 13.75 minutes | |
| --- | --- |
| 1 | Wrist dorsiflexion |
| 2 | Close-legged trunk flexion |
| 3 | Open-legged trunk flexion |
| 4 | Open-legged lateral trunk bending |
| 5 | Cross-legged trunk flexion |
